# Recent advances and future directions in etiopathogenesis and mechanisms of reactive oxygen species in cancer treatment

**DOI:** 10.3389/pore.2023.1611415

**Published:** 2023-10-18

**Authors:** Priyanka Verma, Bhavika Rishi, Noreen Grace George, Neetu Kushwaha, Himanshu Dhandha, Manpreet Kaur, Ankur Jain, Aditi Jain, Sumita Chaudhry, Amitabh Singh, Fouzia Siraj, Aroonima Misra

**Affiliations:** ^1^ Department of Health Research, Indian Council of Medical Research-National Institute of Pathology, New Delhi, India; ^2^ Vardhman Mahavir Medical College and Safdarjung Hospital, New Delhi, India

**Keywords:** reactive oxygen species (ROS), recent advances, future directions, cancer therapy, ferroptosis

## Abstract

A class of exceptionally bioactive molecules known as reactive oxygen species (ROS) have been widely studied in the context of cancer. They play a significant role in the etiopathogenesis for cancer. Implication of ROS in cancer biology is an evolving area, considering the recent advances; insights into their generation, role of genomic and epigenetic regulators for ROS, earlier thought to be a chemical process, with interrelations with cell death pathways- Apoptosis, ferroptosis, necroptosis and autophagy has been explored for newer targets that shift the balance of ROS towards cancer cell death. ROS are signal transducers that induce angiogenesis, invasion, cell migration, and proliferation at low to moderate concentrations and are considered normal by-products of a range of biological activities. Although ROS is known to exist in the oncology domain since time immemorial, its excessive quantities are known to damage organelles, membranes, lipids, proteins, and nucleic acids, resulting in cell death. In the last two decades, numerous studies have demonstrated immunotherapies and other anticancer treatments that modulate ROS levels have promising *in vitro* and *in vivo* effects. This review also explores recent targets for therapeutic interventions in cancer that are based on ROS generation or inhibition to disrupt the cell oxidative stress balance. Examples include-metabolic targets, targeted therapy with biomarkers, natural extracts and nutraceuticals and targets developed in the area of nano medicine. In this review, we present the molecular pathways which can be used to create therapy plans that target cancer by regulating ROS levels, particularly current developments and potential prospects for the effective implementation of ROS-mediated therapies in clinical settings. The recent advances in complex interaction with apoptosis especially ferroptosis and its role in epigenomics and modifications are a new paradigm, to just mechanical action of ROS, as highlighted in this review. Their inhibition by nutraceuticals and natural extracts has been a scientific challenging avenue that is explored. Also, the inhibition of generation of ROS by inhibitors, immune modulators and inhibitors of apoptosis and ferroptosis is explored in this review.

## Introduction

Cancer remains a significant global health burden, affecting both developed and developing nations, despite advancements in cancer diagnosis, treatment, and prevention. Annually, approximately 10 million individuals lose their lives to cancer, while 19–20 million new cancer cases are diagnosed worldwide [1]. ROS are closely linked to a number of malignancies, including cancers of lung, breast, colorectal, cervical, and hepatocellular carcinoma, which are among the most common cancers in terms of incidence cases globally. The etiopathogenesis of cancer is multifaceted [2]. An extensive understanding of the role of ROS in the causes and progression of cancer, along with their function in the progression of the disease, can greatly enhance targeted therapies. The latest advances in understanding the generation of ROS, including role of ferroptosis, epigenetic changes associated with ROS and its etiopathogenesis in cancer and its role in cancer progression is continuously being explored.

**FIGURE 1:**
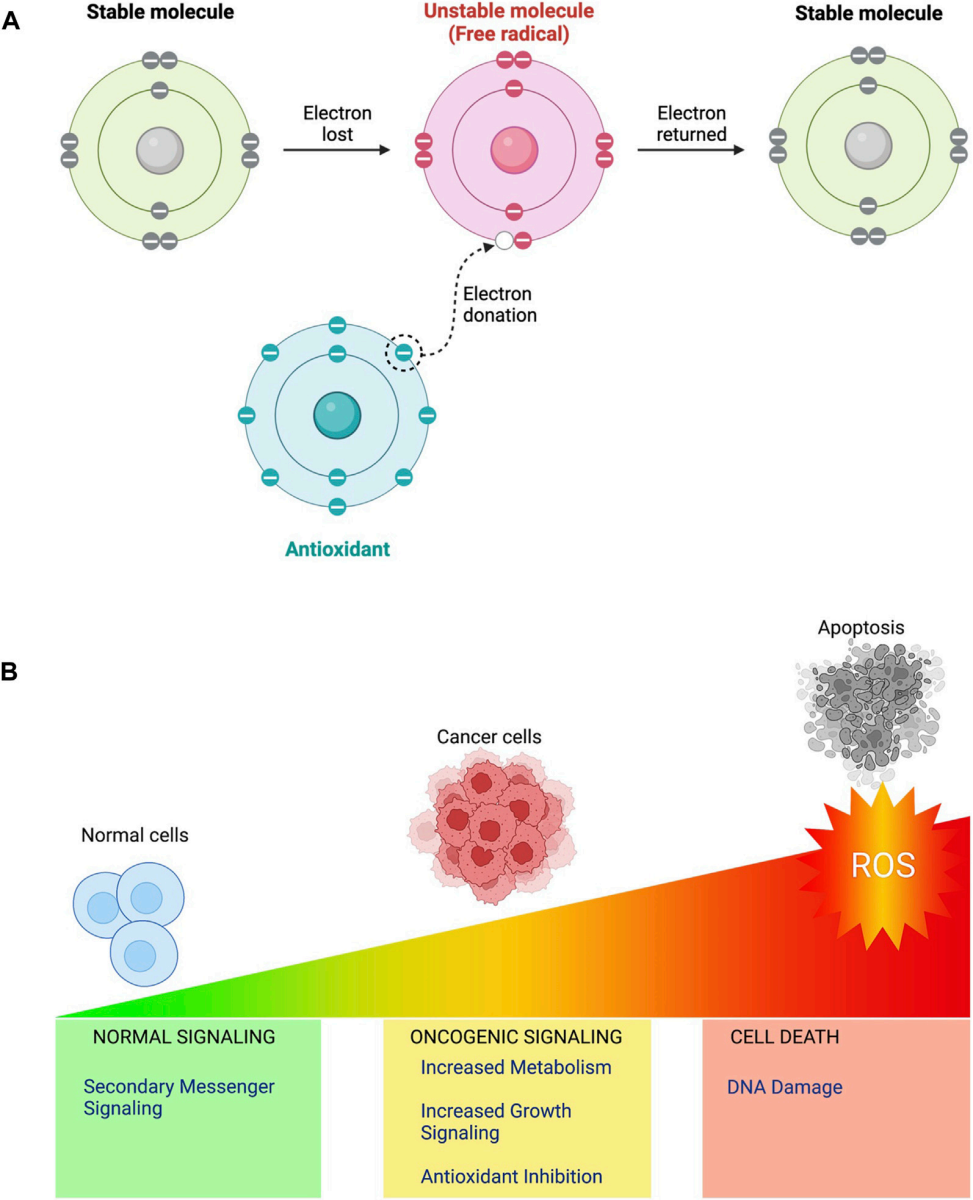
**(A)** When electrons are lost from stable compounds in biological systems, free radicals or ROS are frequently produced. This generated unstable, highly reactive molecules with unpaired electrons are especially reactive because they want to couple their unpaired electrons. Antioxidants are stable compounds which can donate an electron or a hydrogen atom to stabilise a free radical. **(B)** ROS function as intracellular second messengers at low doses and take part in cell communication and control a variety of physiological functions. Moderate ROS levels can inhibit antioxidant activity, limiting the cell’s capacity to combat ROS and contributing to cancer cell survival. Excess ROS can cause oxidative stress, which can damage biological components, including DNA and can activate apoptotic pathways, resulting in programmed cell death (apoptosis).

“ROS” is an umbrella term attributing to unstable, reactive, partially reduced oxygen derivatives generated by oxidation-reduction (redox) or electronic excitation processes [3] (Figure 1A). They are made up of free radicals such as hydroxyl (HO*)and superoxide (O_2_*), in addition to non-radical molecules such as hydrogen peroxide (H_2_O_2_), which are generally less reactive than most ROS but can enter any cellular compartment before they’re broken down into oxygen and water by peroxiredoxins and GPXs (glutathione peroxidases) [3].

H_2_O_2_ contributes to redox signalling by acting as a second messenger in a variety of pathways that influence gene expression and the transmission of external signals [3]. ROS are by-products of several biological activities, including oxygen consumption, playing a crucial role in various biological processes within both healthy and malignant cells [3]. Increasingly evident research indicates that ROS behave in cancer cells in a " double-edged " way [4]. At low to moderate concentrations, reactive oxygen species (ROS) act as signal transducers, stimulating cancer cell motility, drug resistance, angiogenesis, and invasion. On the other hand, high quantities of ROS injure cancer cells and ultimately cause cell death (Figure 1B). Therefore, tight synchronization of ROS levels is essential for cellular life [4]. Thus, antioxidants are important in Redox biology (Figure 1A). Superoxide dismutases (SODs) found in the cytoplasm, mitochondria, and extracellular matrix (ECM), along with glutathione reductase (GR), GPX, thioredoxin, peroxiredoxin and catalase, each function collectively in eukaryotic cells to recycle antioxidants in the reduced state and reduce O_2_* anions into water [5]. In this article, we examine existing cancer therapy approaches that employ ROS pathways and offer insights into their potential as therapies in the future.

## Generation of ROS and redox homeostasis

**FIGURE 2:**
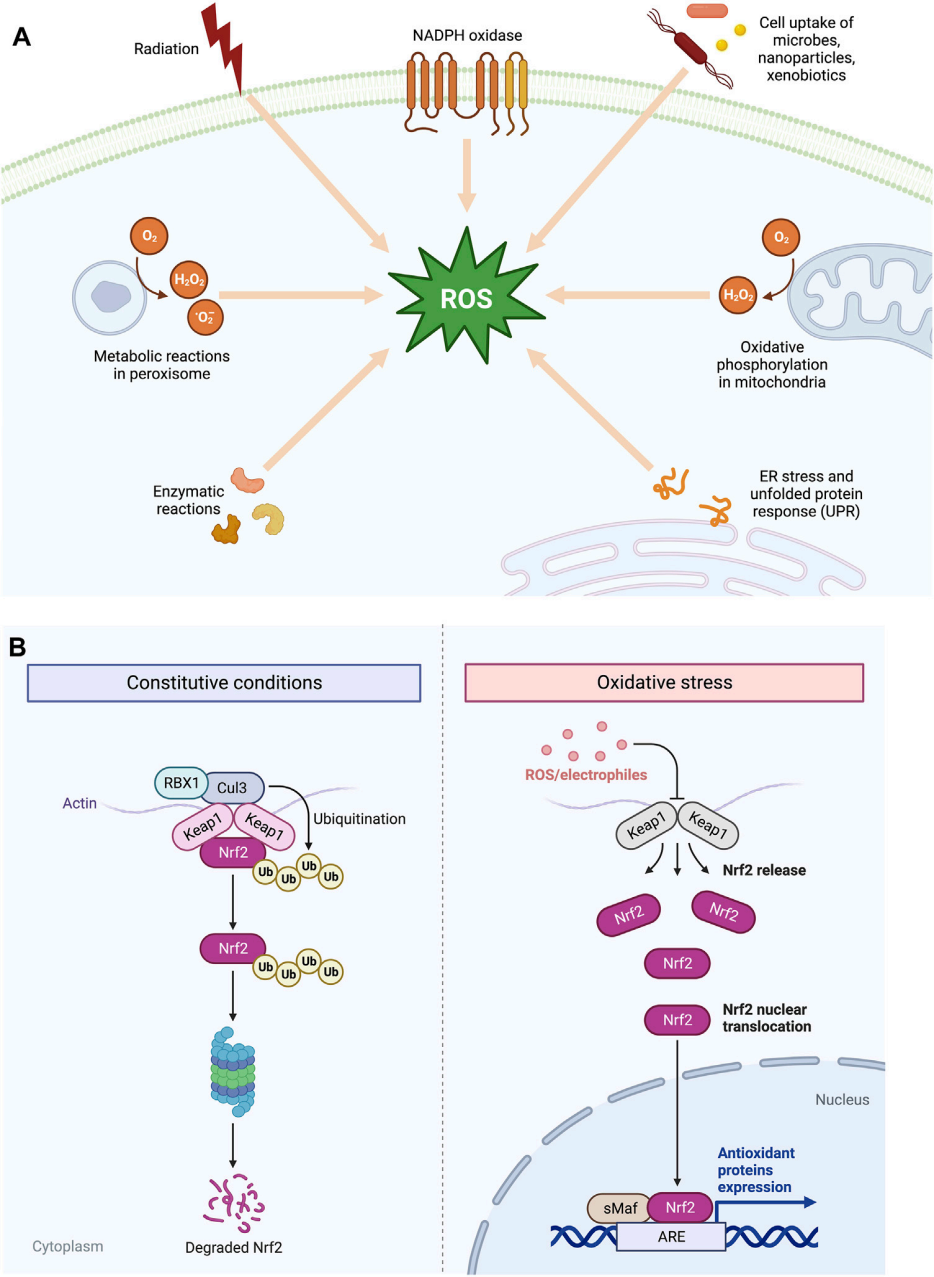
**(A)** Endogenous and exogenous sources of Reactive Oxygen Species (ROS). **(B)** The pathway between KEAP1 and NRF2 is shown schematically. Under normal physiological circumstances, NRF2 is found next to KEAP1 in the cytoplasm to activate Cul3‐dependent ubiquitination and its degradation via the proteasome. Under oxidative stress, NRF2 separates from KEAP1 and moves into the nucleus and activates several cytoprotective genes.

ROS are produced by various exogenous and endogenous sources. The electron transport chain (ETC) and the trans-membrane NADPH oxidases (NOXs) on the inner mitochondrial membrane are the most important endogenous enzymatic producers of O_2_* and HO* free radicals [3, 6]. In addition, oxidants can be produced by peroxisomes and endoplasmic reticulum (ER) and enzymes, including xanthine oxidase, cyclooxygenases, nitric oxide synthase, cytochrome P450 enzymes, and lipoxygenases [2] (Figure 2A). NOXs have many cellular localizations, which contribute to the local generation of ROS and allow H_2_O_2_ compartmentalization for cell signalling via cell surface receptors. The two vital areas for generation of ROS are complexes I and III where the leakage of electrons leads to the generation of O_2_* [4]. While complex III releases its products into the cristae lumen and the intermembrane gap, complex I releases its products into the mitochondrial ETC in the direction of the mitochondrial matrix [6]. Along with complex I and III of mitochondrial ETC as major sources of ROS, downregulation of Complex IV of cytochrome c-oxidase also contributes to the generation of increased levels of mitochondrial and cellular ROS and enhanced glycolysis, as revealed by Agnireddy et al. The Fenton reaction involving Fe^2+^ can also give rise to ROS non-enzymatically. Fe [2] reacts H_2_O_2_ with to yield HO* radicals, which can cause destruction to genome and other biomolecules [6]. Beside intracellular sources, multiple external factors, known as the “exposome,” induce exogenous ROS as a result of accumulative environmental exposure, including molecular components such as nutrients, drugs, pollutants, and toxicants, along with physical stressors such as UV, X-ray, and other ionising radiations, and lifestyle [3, 7]. Therefore, multiple intracellular and external stimuli work together to promote the generation of ROS in tumour cells (Figure 2B).

Apoptosis and cell-cycle arrest are brought on by excessive ROS concentrations. In response to oxidative stress, cancer cells increase the production of antioxidant enzymes as a protective measure against excessive intracellular reactive oxygen species (ROS). The NRF2-KEAP1 complex is an example of a physiological sensor-effector apparatus based on thiols that responds to an oxidant challenge and plays a part in eukaryotic redox homeostasis [4]. It is a key detector for electrophilic and oxidative stressors, and KEAP1 that serves as an NRF2 inhibitor contains many cysteine residues which are vulnerable to oxidation. The oxidation process plays a crucial role in the formation of Cys-disulfide within the KEAP1 dimer. This oxidation induces a conformational change in KEAP1, enhancing its stability and facilitating the transportation of NRF2 to the nucleus. In the nucleus, NRF2 acts as a transcription factor (TF), regulating the expression of various antioxidant defences proteins [4] (Figure 2B).

## ROS interactions with lipids, proteins, genome and epigenome

In ROS synthesis with an increase or decrease in the amount of ROS scavenged, cells experience oxidative stress. Further, elevated intracellular ROS levels may affect proteins lipids, and DNA, and this capacity of ROS has been used in a number of anti-tumor approaches, as mentioned below. ROS have the ability to oxidize intracellular lipids, proteins, and DNA, resulting in the accumulation of damaged biological elements.

### ROS and lipids

One important area of research in ROS is lipid-derived ROS, which is generated when polyunsaturated fatty acids are oxidised and result in lipid H_2_O_2_ and related radicals like peroxyl and alkoxyl [3]. ROS can produce oxidative stress by their interactions with lipids via a feedback loop initiated by fatty acid peroxidation, which changes lipid bilayer of cellular membranes [5]. Such oxidised lipids have an important function in redox signalling, especially in immunological signalling. Examples of enzymes that create reactive oxidants are lipoxygenases and prostaglandin synthases (cyclooxygenases), which operate as mediators in the mechanisms that start and control inflammatory responses. The breakdown of ETC complexes I and III and mitochondrial phospholipids peroxidation, which may potentially affect the integrity of PTPs (permeability transition pores) and enhance electron transport is potentially harmful to the cells [5].

### ROS and proteins

Protein function is affected by redox signalling, which results in alterations in signalling outputs, activity of enzymes, DNA transcription, and membrane and chromosomal integrity [5, 6]. The biological effects of ROS are exerted in redox regulation mostly through thiol-based protein modification. Cysteines are particularly susceptible to oxidation in many proteins, including cytoskeletal components, scaffold proteins like 14-3-3, and, HSPs (heat shock proteins), and various ribonucleoproteins (RNPs), raising the prospect of functional protein networks controlled by redox [3]. Since the by-product of protein folding is H_2_O_2_, ROS are crucial to ER stress and protein folding [3]. ROS by interacting with proteins impacts signaling pathways implicated in the regulated cell expansion and apoptosis control. They tend to inhibit phosphatases whereas kinases can either be activated or inhibited. The Src family of nonreceptor protein kinases, small G proteins like Ras, growth factor tyrosine kinase receptors, and elements of the c-Jun p38 kinase (p38MAPK) and c-Jun N-terminal kinase (JNK) pathways that trigger apoptosis are all particularly activated by ROS [5, 8]. Small rise in ROS are predicted to preferentially trigger the PI3-K/Akt pathway, whereas larger levels are predicted to cause p38MAPK-dependent apoptosis [8]. Additionally, ROS affect calcium channel function. They cause the release of calcium from cellular reserves, which in turn activates kinases like protein kinase C, and is a key factor in the growth of cancer cells [5].

### ROS interactions with genome

Single-strand and double-strand breaks, base modifications, deoxyribose alterations, and DNA cross-linking are all examples of DNA damage brought on by ROS. Finally, ROS has a direct impact on TFs’ capacity to bind DNA [2]. For instance, ROS boost the nucleus localization of the Fos/Jun DNA-binding protein redox factor-1 (Ref-1), and the ataxia-telangiectasia mutated (ATM) serine/threonine kinase, which is responsible for DNA damage repair [5].

To avoid excessive ROS production, cancer cells activate the transcription of antioxidant enzymes, demonstrating usefulness of a comprehensive understanding of these networks in unravelling the treatments responsible for lowering ROS levels. When ROS levels rise, JNK activates the forkhead box O (FOXO) family of TFs, which stimulates the synthesis of SODs and catalase. Despite the fact that the H_2_O_2_ produced by SODs from O* serves as a substrate for catalase, activation of SODs by a FOXO4 TF appears to counteract their antioxidant effect [5]. The expression of the antioxidant gene is significantly regulated by p53, another significant TF is inhibited by moderately raised ROS levels, but its expression is encouraged by greater ROS levels [5]. Further, ROS can activate or impede NFκB role depending on the context. By oxidizing and activating the inhibitor of NFκB (IκB) kinases, which inhibit IκB stability, cytosolic H_2_O_2_ can activate the NFκB pathway. Since the DNA binding domain of NFκB contains oxidizable cysteines, H_2_O_2_ can also directly influence its activity. During this situation, increasing nuclear H_2_O_2_ production hampered NFκB DNA binding and decreased transcriptional activity, but increasing nuclear levels of the H_2_O_2_ scavenger peroxiredoxin 1 boosted transcriptional activity [3].

When ROS levels are moderate, they activate HIF1 (Hypoxia Inducing Factor) which results in the activation of numerous genes crucial for the growth of cancer, including VEGF and VEGF receptors [3].

### ROS interactions with epigenome

ROS impact the functioning of epigenetic modifiers such as DNA methyltransferases (DNMTs) and histone deacetylases (HDACs) with repercussions for target gene expression and can result in both hypomethylation and hypermethylation of DNA [9]. They oxidise DNA as well, particularly guanine and adenine (8-oxo-G and 8-oxo-A). It has been discovered among every 100,000 guanines in regular cells, a portion undergoes oxidation, and this proportion rises by 35%–50% in cancer cells [3]. Unrepaired 8-oxo-G is highly mutagenic, particularly when it interacts with A, leading to G to T transversions. Due to these characteristics, it holds significant potential as an indicator of mutagenesis and cancer triggered by reactive oxygen species (ROS). Furthermore, 8-oxo-Gs build up at telomeres, inhibiting telomerase and reduce telomeric protein binding, altering telomere length and impeding chromosomal-end capping maintenance [3, 10]. Other sorts of oxidative damage can cause DNA demethylation like ROS hydroxylate methylcytosine to produce 5-hydroxymethylcytosine (5hmC) [6]. A feedback loop potentially resulting from alterations in mitochondrial DNA caused by ROS, results in mutations in genes producing ETC complexes that may significantly impair electron transport efficiency.

## Heterogenous function of ROS in cancer

The world’s second-leading cause of death is Cancer, and it is distinguished by a number of hallmarks, which includes cell transformation, DNA instability, angiogenesis, hyper growth, epithelial-mesenchymal transition (EMT), immortalization, and metastasis, all of these are regulated in a variety of manners by intracellular ROS. To develop more effective cancer therapies, the mechanism of ROS in carcinogenesis must be investigated. When ROS levels surpass normal levels, they can trigger carcinogenesis and accelerate tumour growth. Excessive ROS production can lead to genetic mutations, which stimulate oncogenes and block tumour suppressor genes including KRAS oncogene and p53, resulting in cancer development [2]. Raised ROS additionally boosts tumour formation by lowering the activity of NK cells and T cells and increasing macrophage engagement and M2 polarization. Furthermore, high levels of ROS encouraged tumour invasion and metastasis mediated EMT [2]. As discussed in Section *ROS interactions with epigenome*, the buildup of 8-oxodG in cellular genomes causes cancer. It is noteworthy that iron-mediated oxidative stress is a predictor underlying emergence of several malignancies. Cancer-associated fibroblasts (CAFs), which are especially abundant in tumour microenvironment (TME), contribute actively to the control of tumour homeostasis, encouraging tumour growth and cancer cell migration [2].

ROS inhibit the proliferation of cancer cells, hence preventing their expansion. ROS directly reduce EGF and EGFR expression leading to blockage of downstream signaling molecules responsible for proliferation including ERK and PI3K/Akt. H_2_O_2_ injection can inhibit ERK1/2 phosphorylation in breast cancer cells, slowing the growth of cancer cells dependent on its dosage [11]. Additionally, high ROS prevent androgen-independent prostate cancer cell growth by preventing EGF-triggered EGFR-mediated PI3K/Akt signaling. Additionally, ROS interfere with the PI3K/Akt/NFκB signaling network, preventing the growth of A549 human lung cancer cell [2]. ROS buildup in multiple myeloma cells decreases phosphorylation of Src and Janus kinases (JAK1, JAK2), obstructing STAT3/STAT5 phosphorylation and reducing the transcription of cyclins (B1, D1 and E) and cyclin dependent kinases (CDK2,CDK4) ultimately resulting in arresting of cell cycle and slowing cancer growth [2]. Moreover, the buildup of ROS regulates molecules that govern the cell cycle, including the CDC25 (cell division cycle) and CDK inhibitors. CDC25B is rendered inactive by LGH00031, which then dephosphorylates CDK1’s tyrosine to stimulate the CDK1/cyclin B complex, causing the cell cycle to be arrested and the proliferation of cancer cells to be inhibited [12]. Highly mitotic cells require an adequate supply of nucleotides and ATP. As a result of the buildup of ROS, cancer cells may be unable to produce enough ATP and nucleotides to support cell growth [2].

## ROS induced programmed cell death

### ROS and apoptosis

**FIGURE 3:**
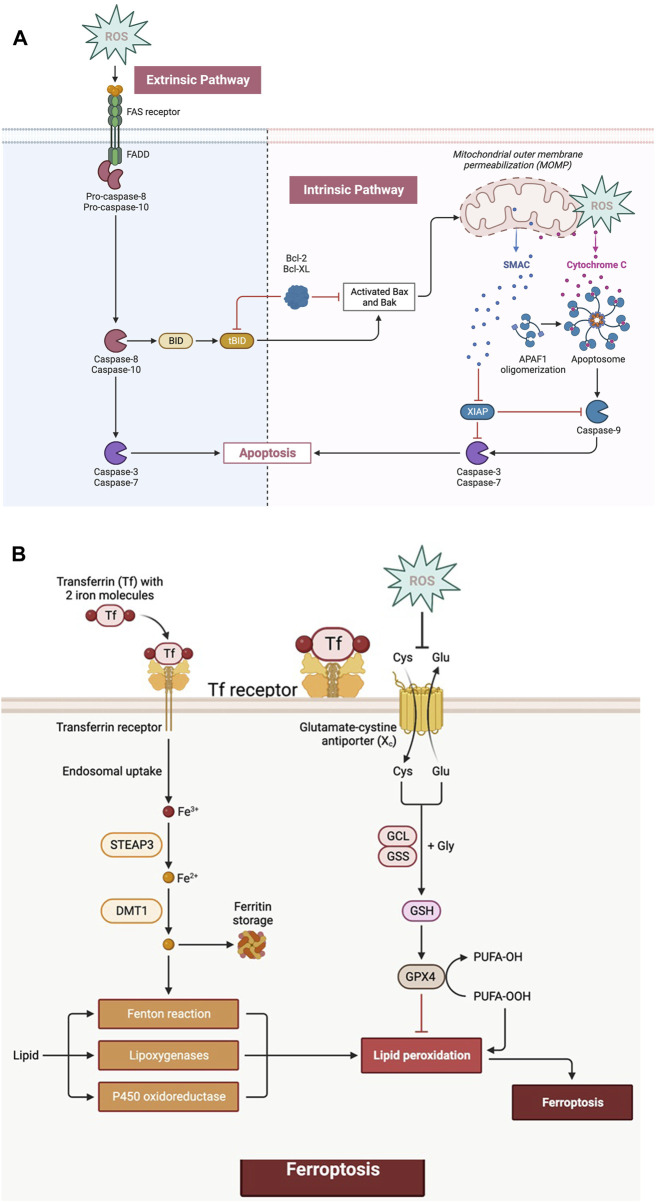
**(A)** ROS can increase death receptor clustering and aggregation on the cell membrane, boosting their signalling potential and extrinsic pathway. The activation of initiator caspases subsequently activate downstream effector caspases, leading to apoptosis. Intracellular stress signals activate the intrinsic route, also known as the mitochondrial pathway. Permeabilization of the mitochondrial membrane allows for the release of cytochrome c from the mitochondria, which, together with other components, forms the apoptosome, which activates the caspases leading to apoptosis. **(B)** The absorption of extracellular iron by particular transporters, such as transferrin receptor 1 (TfR1), initiates ferroptosis. The free iron in the cytoplasm can participate in Fenton reactions, which produce highly reactive OH* radicals. Furthermore, lipid metabolism in cells generates lipid peroxides (LOOH) which react with polyunsaturated fatty acids (PUFAs), causing lipid peroxidation. During ferroptosis, there is a significant depletion of GSH due to a decrease in cystine absorption, and the activity of antioxidant enzymes such as GPX4 is reduced, resulting in increased oxidative stress.

Higher concentrations of ROS either intrinsically or extrinsically control the “apoptosis” process and H_2_O_2_ is one of the most significant ROS in terms of its ability to directly and powerfully cause apoptosis. ROS interacts with mitochondrial ETC, causing cytochrome c release by rupturing the mitochondrial membrane and opening PTP. Cytochrome-c, in collaboration with Apaf-1 (apoptotic peptidase activating factor 1) and procaspase-9, generates “apoptosomes” in the cytosol that activate caspase-9, which in turn triggers effector caspases such as caspase-3 or 7, culminating in protein breakdown and apoptotic cell death [5] (Figure 3A). The extrinsic route is activated by death-inducing ligands binding to cognate receptors, such as Fas ligands and TNF-related apoptosis-inducing ligands (TRAIL-R1/2) which in turn attract pro-caspases and adaptor proteins. As a result, the death-inducing signalling complex (DISC) forms, effector caspases are activated, and apoptosis is triggered [4]. Additionally, Caspase-8 and Caspase-10 cleave Bid to create truncated Bid (tBid), which translocates to mitochondria, inhibits BcL-2 and BcL-xL’s anti-apoptotic activity and activates Bax and Bak [4] (Figure 3A). Additionally, the generation of oxidative stress in mitochondria is necessary for the activation of the p53-induced intrinsic apoptosis signalling pathway [13]. The elevated ROS level influences p53’s translational modification and stabilisation, which activates p21-mediated cell cycle arrest [13].

Chemotherapy and radiation generate intracellular ROS, which can employ intrinsic or extrinsic routes to trigger apoptosis. Procarbazine generates oxidative damage of DNA that is not mended by the BER/NER system in brain tumours and Hodgkin’s lymphoma. ROS production during doxorubicin-relied cytotoxicity is successfully utilised to treat, breast cancer, Kaposi’s sarcoma and acute lymphocytic leukaemia (ALL) [5, 14]. Apoptosis has been demonstrated to be triggered by a course of therapy that includes the DNA replication-impairing agent arabinocytosine, followed by the ROS-increasing agent anthracyclines, and has been proven to be advantageous for AML patients [15]. Furthermore, the administration of platinum-based medications together with poly ADP-ribose polymerase (PARP) inhibitors, a protein indulged in maintaining DNA integrity, has demonstrated in BRCA-deficient models as well to stop the development of breast cancer cells, raising ROS levels that stimulate apoptosis [16]. Increased ROS on sphingomyelinase, creates ceramide from sphingomyelin and leads to binding to death receptors on cancer cell membranes, may also be an apoptosis mediator [5, 13]. Likewise employing medications that impact mitochondria, which create more than half of all ROS, is an efficient strategy to induce oxidative stress and death in cancer cells [5]. Several medications have been developed to escalate ER stress in cancer cells by inducing oxidative stress. These include celecoxib, an anti-inflammatory drug, which exacerbates ER stress and promotes apoptosis by changing ratio of Bax/BcL-2 and elevating ROS in prostate cancer cells, and bortezomib, a proteasome inhibitor, which leads to ER stress via ROS in squamous carcinoma cells of the head and neck [5].

### ROS and autophagy

“Self-eating” or autophagy is a tightly regulated process that eliminates dysregulated or harmed intracellular proteins. Recently, ROS-induced autophagy has been introduced as a significant therapeutic strategy to eradicate cancer cells. In particular, it has been reported that LC3-associated autophagosomes are increased by H_2_O_2_-dependent inactivation of the autophagy-related gene-4 (ATG4) and that the critical autophagy inhibitor mTORC1 is downregulated by ATM-mediated oxidation of AMPK (AMP-activated protein kinase) [13]. It has been shown that polycyclic ammonium ion sanguinarine therapy, that boosts electron leakage from mitochondria and produces NOXs, causes H_2_O_2_ to cause autophagy in glioma cells [17]. When rapamycin and HSP90 inhibitors are used together, they produce mitochondrial damage, oxidative stress, and autophagy, which slows the development of RAS-dependent tumours [17]. ROS can also regulate autophagy via the stimulation of TF such as NFκB which modulates the transcription of autophagy inducing genes like BECLIN1/ATG6 or SQSTM1/p62 in tumour cells [7].

### ROS and necroptosis

Necroptosis is regulated necrosis mediated by death receptors. It exhibits morphological features of both apoptosis and necrosis and requires protein RIPK3 (receptor-interacting protein kinase 1) and its substrate MLKL, the crucial players of this pathway [7]. ROS can also induce necrosis. According to certain studies, ROS produced following ceramide production or after a rise in energy metabolism both by NOXs or in the mitochondrial ETC have been shown to promote necroptosis [7].

### ROS and ferroptosis

One of the most crucial ways to prevent cancer is through ROS-induced ferroptosis. A very fascinating ROS-associated mechanism of p53’s tumour regression has newly come to light that causes ferroptosis (dependent on the existence of intracellular iron) by increasing ROS levels [18]. In the investigation by Yang et al., several ferroptosis-inducing substances (FINs) were used, and it was discovered that every FIN inhibited GPX4 either directly or indirectly by depleting GSH [19, 20]. They concluded that the main regulator of ferroptosis is GPX4. Phospholipid hydroperoxides (PLOOH) are reduced into their corresponding phospholipid alcohols by the catalytic action of GPX4. For GPX4 to operate normally physiologically, GSH is required. Glutamate-cysteine ligase catalyses the formation of intracellular GSH. Thus, cysteine is a rate-limiting amino acid for GSH synthesis, and it is absorbed as cystine via the cystine/glutamate antiporter (Xc) system. Due to build-up of PLOOH caused by the inactivation of GPX4, cell membrane damage and ferroptotic death may result. It was also shown in Yang et al.'s work that genetically inhibiting GPX4 can cause tumour cell ferroptosis and stop tumour development *in vivo* [20]. Ferroptosis, an iron-dependent kind of cell death, is characterised by an increase in the labile iron pool (LIP), a tiny pool of Fe2+ [21]. The main mechanisms by which cellular iron absorption is mediated is through the binding of transferrin to its receptor, was discovered in 1997. Alternatively, elevated intracellular LIP can contribute to the phospholipids peroxidation to form PLOOH and can produce free radicals via the Fenton reaction. Alternatively, iron catalyses the majority of ROS production in cells. The production of ROS starts lipid peroxidation, which ultimately results in ferroptosis. It has been demonstrated that cancer cells require more iron to survive than healthy ones. Rapidly reproducing cancer cells have increased iron intake and intracellular iron levels, raising the possibility that ferroptosis induction might be used as a therapeutic target for cancer [18]. According to research by Alvarez et al., raising intracellular LIP in lung cancer cells by inhibiting NFS1 makes them more susceptible to ferroptosis and slows the development *in vivo*. Lipoxygenases and cytochrome P450 oxidoreductase (POR) can also be involved in the synthesis of PLOOH during enzymatic lipid peroxidation. The deoxygenation of free and esterified PUFAs by lipooxygenases, nonheme iron-containing enzymes, results in PLOOH [19]. The classical GPX4-controlled route, the lipid metabolism pathway, and the iron metabolism pathway are the three primary avenues to reverse chemotherapy resistance, according to the principles driving ferroptosis [19]. Erastin is a synthetic medication that causes tumour cells with mutant RAS to undergo ferroptosis and by changing the outer mitochondrial membrane’s permeability and raising intracellular ROS levels [18]. According to studies, synthetic medicines like artesunate, sorafenib, and erastin suppress the production of glutathione (GSH), inactivating GPX, which causes oxidative stress in cancer cells and eventually results in ferroptosis [18] (Figure 3B). Through genome-wide, CRISPR-Cas9-mediated suppressor screens, Zou et al. investigated POR as a requirement for ferroptotic cell death in cancer cells [22]. High hopes have been placed on ferroptosis as a novel, promising means of eliminating cancer cells because of ferroptosis inducers.

## ROS in cancer prevention and therapy

### Role of ROS in tumor immunity

In the TME, controlling ROS levels have been demonstrated to have anti-tumour effects. Their response promote cancer cell apoptosis, impede angiogenesis, prevent immune escape, alter tumour metabolic reorganisation, and improve drug resistance. T regulatory (Treg) cells are attracted and act as potent immunosuppressors in TME [23]. It is known in general that the TME, which is constructed of a vast range of cell types, including cancer cells, fibroblasts linked with cancer, and immune cells, produces a region with significant concentration of ROS with high ROS level as the main components responsible for TME’s resistance to immunotherapies, particularly immune checkpoint blockades [23]. Due to the increased ROS concentration present in the TME, tumour-infiltrating Treg cells die. It is noteworthy that tumour-infiltrating apoptotic Treg cells inhibit programmed death-ligand 1-blockade-mediated anti-tumour T cell immunity by switching ATP to adenosine, despite their poor NRF2-associated antioxidant system making them particularly susceptible to ROS [5]. Based on this scenario, the ROS reduction in TME can enhance the effectiveness of cancer immunotherapies. Precisely, by removing ROS, a novel nano-scavenger anchoring on the ECM alleviated suppressive immunogenic cell death [5, 23].

In addition to Treg cells, ROS are important metabolic regulators of cytotoxic T cells and Dendritic cell (DC) mediated anti-tumour immunity [5]. In general, the link between ROS and cancer immunity remains a mystery, which emphasises the ongoing need to research this to increase the effectiveness of immunotherapies.

### Antioxidant nutraceuticals in cancer prevention

Antioxidant nutraceuticals are a grouping of nutraceuticals (a combination of nutrition and medicines) that have antioxidant properties. Numerous antioxidant substances and enzymes in our body control the amount of ROS. In addition to vitamins and minerals like zinc and selenium, it also contains enzymes like GPXs and catalases, whose primary goal is to scavenge ROS. The consumption of naturally occurring antioxidant-rich foods has been suggested as one of the best strategies to ward off cancer. It has been discovered that many nutrients, including vitamins A, C, D, and E, choline (the richest dietary sources of choline are meat, fish, dairy, and eggs) and theanine, have potent antioxidant properties that inhibit the growth of cancer stem cells and tumorigenesis in a variety of tumour types, including breast, lung, brain, and others [24]. Vitamin A can mix with peroxyl radicals and function as a chain-breaking antioxidant before the peroxyl radicals can interact with lipids and produce hydroperoxides, avoiding cellular damage [22]. Supplemental 25-hydroxyvitamin D (Vitamin D) has been shown to lower breast and colorectal cancer death rates while having the reverse impact on prostate cancer [24, 25]. Additionally, it has been shown that enhanced absorption of DHA, an oxidised version of vitamin C, showed preferential death of colorectal cancer cells with KRAS or BRAF mutations through the glucose transporter 1 (GLUT1) pathway [26]. Thyroid cancer cell growth is suppressed by vitamin C, which has been shown to increase the formation of ROS and limit the ERK phosphorylation by reducing EGF release and phosphorylation of its receptor [27]. The class of fat-soluble antioxidant compounds known as vitamin E includes tocopherols and tocotrienols that lower ROS, inhibits the growth of tumours and carcinogenesis, and encourage the death of cancer cells [28]. The few therapies currently available for treating pancreatic cancer by increasing intracellular ROS levels to promote apoptosis are gemcitabine, BITC (Benzyl isothiocyanate) and capsaicin [7]. Aminoflavone (AF), a synthetic substance related to flavonoids, has shown antiproliferative action against many cell lines from the kidney, breast, and ovary. MCF7 and MDA-MB231 cells are killed by AF, whereas normal, non-malignant breast cells are unaffected [7, 27]. An increase in intracellular ROS is seen following AF therapy, coinciding with a rise in the activation of Caspase 3 following apoptosis [29]. Similar to AF, many substances, including IOA, pancratistatin, and Triphala, cause the death of breast cancer cells by raising intracellular ROS concentration through the dissipation of the membrane potential of mitochondria [7].

### Phytochemicals and natural extracts with anticancer properties

Plant-derived chemicals including polyphenols and flavonoids have been demonstrated to be particularly beneficial in the treatment of cancer, with their effect being mediated through scavenging ROS [30]. Strong metal ion chelators and tea polyphenols limit the liberation of ROS from the auto-oxidation of numerous substances. EGCG is the tea catechin that reacts with the majority of ROS most effectively [31]. In HepG2 (hepatic cancer), PC-3 (prostate cancer), and MCF-7 (breast cancer) cells, it has been found that the natural polyphenol resveratrol induces apoptosis through regulating antioxidant enzymes [32]. Further, lower quantities of curcumin (isolated from the dried root of rhizome Curcuma Longa) have been linked to decreased ROS generation, but greater concentrations of curcumin have been linked to increased ROS levels in solid tumours and leukaemia [23]. In the transgenic prostate cancer model, nimbolide was found to create oxidative stress, which delayed tumour development via STAT3 signalling [33, 34]. By preventing ROS from activating MAPKs, β-caryophyllene oxide has been proven to inhibit tumour development and promote apoptosis [30]. The main bioactive component of cayenne peppers is capsaicin, a homovanillic acid derivative [35] causes redox imbalance and ferroptosis in glioblastoma cells via ACSL4/GPx4 signalling pathways [36]. Piperine administration lowered mitochondrial lipid peroxidation caused by oxidative stress in mice with lung cancer and improved the activity of both the enzymatic (catalase, SOD, and GPX) and non-enzymatic (reduced GSH, vitamin C and vitamin E) anti-oxidant defence system [37]. Koumine, a common alkaloid monomer in *Gelsemium* plants, has a strong antioxidant impact which prevents ERK from being phosphorylated and, as a result, slows the growth of HCCs [38]. Genistein, a particular class of isoflavone occurs naturally in soy and soy products [39] acts as an inhibitor of radiation- and carcinogen-induced tumours of the liver and mammary glands due to its antioxidant and antiproliferative properties. The scavenging of ROS, the prevention of oxidative and photodynamic DNA damage, and the inhibition of tyrosine protein kinase are some of the potential mechanisms behind genistein’s anticarcinogenic effects [39, 40]. In addition, the phenol thymol causes human glioblastoma cells to undergo both apoptosis and necrosis. Thymol stimulates the ROS formation, which causes DNA damage and cell membrane rupturing in addition to having lethal actions on many malignant cells [34]. The monoterpene compound thymoquinone, the primary essential oil component of *N. sativa* seeds [34], inhibits the PI3K/Akt signalling network in bladder and breast malignancies while activating ROS production to phosphorylate p38 [40].

The anticancer efficacy of plant extracts against various cancer cell lines that have been examined *in vitro* and *in vivo*. Some plant metabolites that have been identified and extracted have distinctive bioactivities that improve therapeutic efficacy through ROS-mediated mechanisms [30]. SW480 (human colorectal cancer cells) underwent cell growth arrest and apoptosis when treated *with M. alba* root bark extract. In this investigation, the extract showed that cyclin D1 was degraded by the proteasome and that ATF3 was activated by ROS and GSK3-dependent signalling. Additionally, the growth of the Calu-6 (pulmonary), HCT-116 (colon) and MCF-7 (breast) cancer cell lines was suppressed by a methanolic extract of *M. alba* leaves [30]. The pharmacological effects of platycodin D, a significant triterpenoid saponin isolated from *P. grandiflorus* roots, including its anti-tumour, anti-inflammatory, anti-obesity, and antiallergy properties, have also been thoroughly researched. Platypodin D activated the Egr-1 gene in human leukemic U937 cells, resulting in eventual generation of ROS that induced apoptosis and cell death [30].

### Role of ROS in nanomaterial-mediated targeted cancer therapy

With the exceptional qualities of nanoparticles, like strong biocompatibility, favourable pharmacological parameters, inherent targeting capabilities, and perfect physical and chemical attributes, nanotechnology has spurred significant advances in medicine during the last decades [41]. Nanomaterials’ physicochemical features, such as size and charge, make them highly penetrating, allowing them to be targeted at the tumor location. By targeting ROS metabolic routes, ROS-based nanoparticulate enhance ROS production intracellularly, eventually triggering cancer cell death. ROS-producing nanoparticles can be triggered in several ways, involving sonodynamic therapy, photodynamic treatment, chemodynamic therapy, and chemically administered ROS inducers [42]. Chemodynamic treatment is activated by the Fenton reaction, which uses endogenous H_2_O_2_ and metal ions like iron and copper. Photodynamic therapy is caused by an internalized photosensitizer agent that is activated by light, whereas sonodynamic treatment is caused by very penetrating acoustic waves that induce a family of sound-responsive sonosensitizers [42]. These therapy techniques can cause electron transport to the surrounding environment and the generation of ROS. Metal-based NPs such as iron, gold, copper, cerium or titanium, and organic nanoparticles employed as PSs or sonosensitizers, nanoscale DDSs (drug delivery systems), with small molecules carrying chemically encapsulated medicines are examples of ROS-based nanomaterials [42]. So far, protein-based nanomedicine for anticancer therapy has a large family of members, even though the proteins employed in nanomedicine construction frequently vary in their operative activities [43]. Since mitochondria play an important function in tumor formation and development, mitochondria-targeting nanotechnology may be a potential technique for next-generation cancer treatment. The principal generator of ROS is mitochondrion. Huang et al. described the use of mitochondria-targeted hybrid nanozymes as superoxide scavengers to safeguard mitochondria from oxidative damage [44]. They developed a biomimetic nanozyme using ferritin-heavy-chain protein and a metal nanoparticle core. Animal investigations showed that the synthesized nanonzyme has superoxide dismutase and catalase-like capabilities and targets mitochondria by curbing numerous biological barriers, providing an alternative route for regenerative medicine to alleviate oxidative damage. As a result, it was plausible to assume that mitochondrial ROS modulation may be an excellent therapeutic method for cancer treatment [45].

## Conclusion and future perspectives

Given the importance of ROS in tumour cell adaptability, it has been suggested that administration of antioxidants like vitamin E or C could be an approach to avoid malignant conversion. Ferroptosis is expected to play a significant role in modern anticancer medicines, and targeting the apoptotic process is a frequent strategy in cancer therapy. The primary barriers to treating cancer are intrinsic and acquired resistance. According to certain reports, tumour cells may dramatically strengthen their ability to protect against oxidative stress by adversely regulating ferroptosis, which results in resistant survival. Nanomaterials that produce deadly ROS selectively in tumour cells have been developed as a result of breakthroughs in nanotechnology used in cancer therapeutic applications. The advantages of ROS scavenging techniques for cancer avoidance, on the other hand, remain contradictory. Many cancers enhance the activity of antioxidant systems, implying that a delicate balance of antioxidants and oxidants is required for cancer. Cancer cells’ high antioxidant capacity is intermittently associated to chemotherapy resistance, as a number of anticancer medications currently in use are recognized for inducing cytotoxicity to varying degrees by producing oxidants. Plant extracts include numerous active substances such as alkaloids, terpenes, flavonoids, steroids, saponins, and glycosides; the mechanisms and therapeutic effects are the integrated impact of their separate activities that are synergistic, antagonistic, or neutralised. Therefore, to determine and identify the minimum effective dosage and maximum acceptable dose of a specific sample, toxicity studies are necessary. Predominantly for effective anticancer therapy, the use of oxidant generating chemotherapeutics to activate antioxidants or oxidative stress to disrupt the redox balance required for tumour development should be customized to the specific condition, taking into account the degree and type of cancer, oxidant levels in the tumour environment, and the tumour’s internal antioxidant competence. Considering the most recent established methods for analysing specific oxidants, an important way ahead for future investigations to comprehend the complexities of oxidant contributions to physiology is to limit the study of “ROS,” which does not represent one molecule after all, *in lieu* of examining unique molecular drivers like H_2_O_2_* or O_2_*, which have completely distinct characteristics, mechanisms, and impact on physiology and may necessitate entirely distinct methodologies. Furthermore, it is important to better characterise the context-dependent boundary between oxidative distress and oxidative eustress in various physiological contexts. Therefore, it is necessary to characterise redox balance in various circumstances to effectively apply redox regulation as a therapeutic strategy. Access to comprehending such global consequences will be made possible by genomics and imaging technology. We believe that these developments will help the developing area of redox medicine reach its full potential.
